# Self and microbiota-derived epitopes induce CD4^+^ T cell anergy and conversion into CD4^+^Foxp3^+^ regulatory cells

**DOI:** 10.1038/s41385-020-00349-4

**Published:** 2020-11-02

**Authors:** Michal P. Kuczma, Edyta A. Szurek, Anna Cebula, Vu L. Ngo, Maciej Pietrzak, Piotr Kraj, Timothy L. Denning, Leszek Ignatowicz

**Affiliations:** 1 Institute for Biomedical Sciences, Georgia State University, Atlanta, GA USA; 2 Mathematical Biosciences Institute, Ohio State University, Columbus, OH USA; 3 Department of Biological Sciences, Old Dominion University, Norfolk, VA USA

## Abstract

The physiological role of T cell anergy induction as a key mechanism supporting self-tolerance remains undefined, and natural antigens that induce anergy are largely unknown. In this report, we used TCR sequencing to show that the recruitment of CD4^+^CD44^+^Foxp3^−^CD73^+^FR4^+^ anergic (Tan) cells expands the CD4^+^Foxp3^+^ (Tregs) repertoire. Next, we report that blockade in peripherally-induced Tregs (pTregs) formation due to mutation in CNS1 region of Foxp3 or chronic exposure to a selecting self-peptide result in an accumulation of Tan cells. Finally, we show that microbial antigens from *Akkermansia muciniphila* commensal bacteria can induce anergy and drive conversion of naive CD4^+^CD44^-^Foxp3^−^ T (Tn) cells to the Treg lineage. Overall, data presented here suggest that Tan induction helps the Treg repertoire to become optimally balanced to provide tolerance toward ubiquitous and microbiome-derived epitopes, improving host ability to avert systemic autoimmunity and intestinal inflammation.

## Introduction

T cells anergy is a long-term, but reversible, state of unresponsiveness acquired by naive T cells (Tn) upon suboptimal activation by cognate MHC/peptide complexes that occurred in the absence of co-stimulatory signal^1^. Alternatively, anergy can be also induced in potentially autoreactive CD4^+^Foxp3^−^ T cells upon binding of the inhibitory receptors PD-1 or CTLA4 by regulatory CD4^+^Foxp3^+^cells (Tregs)^2^. Mechanistically, anergy results from a transcriptional silencing of activation inducible genes, which is reinforced by epigenetic modifications negatively regulating TCR- signal transduction. Altered mTORC1 and Ras/MAPKs signaling in addition to NFAT homodimer formation are initial intracellular events that recruit histone deacetylases, activate Egr2/3, Sirt and Ikaros transcription factors and redistribute Cbl-b and Itch E3 ligases from the cytosol into endosomes. These changes repress cytokine production and expression of phospholipase C-γ1 and PKC-θ, which ultimately leads to proliferative arrest in anergic T cells^3,4^.

Recently, it has also been shown that a significant portion of anergic T cells converts to Tregs when transferred to lymphopenic hosts, demonstrating that the former subset constitutes a major reservoir of Treg cell precursors^5^. Thus, anergy induction has been described as an “infectious tolerance” mechanism in which a small number of Tregs exerts tolerance by inducing anergy in naive and effector CD4^+^ cells, of which only a fraction differentiates to peripherally derived Treg (pTregs) cells^6,7^. In lymphopenic conditions, Tregs absence prevents the induction of anergy in transferred, naive CD4^+^CD45RB^high^ cells that become effectors activated by microbiota-derived antigens, and ultimately cause wasting disease. In contrast, when lymphopenic mice receive an adoptive transfer of CD4^+^Foxp3^−^ Tan cells, these recipients do not succumb to wasting disease because the fraction of the transferred subset has been already committed to converting to Tregs^5^.

Reportedly, in healthy mice, pTregs originating from anergic precursors help to control multiple autoimmune diseases including diabetes, arthritis, and gastritis demonstrating that long-term maintenance of viable anergic T cells not only supports pTregs conversion but also directly helps sustain tolerance^6,8,9^. Anergic cells have been discriminated based on high expression of FR4^+^CD73^+^PD-1^+^, ubiquitin ligases GRAIL, Cbl-b and Itch, and elevated levels of Nrp1, CD69, Nur77, CD5^5^. High expression of these markers may result from increased self-reactivities of these cells, which also drives their conversion to pTregs^10^. The mechanism(s) controlling the conversion of some Tan cells to pTregs remains incompletely understood, although it involves partial demethylation of the Foxp3 CNS2 region^4^. Differentiation of anergic CD4^+^Foxp3^−^ cells to pTregs proceeds in mice housed in gnotobiotic or SPF facilities, but the SPF strains have an overall higher number of pTregs in their colons^10^. These observations suggest that both tissue and microbiota-derived antigens support conversion of Tn cells to FR4^+^CD73^+^PD-1^hi^ Tan cells, with the latter set of antigens primally impacting mucosal pTregs formation.

In this report, we examined how induction of anergy in CD4^+^ T cells results from an encounter with ubiquitously expressed self-antigen derived from the body’s tissues or microbiota-derived antigens originating from the intestinal microbiota. We show using mice that express class II MHC molecules covalently bound with only a single autoantigen have an elevated number of anergic CD4^+^ T cells, despite exclusive contact with the original selecting self-peptide. Thus, constant exposure of specific CD4^+^ T cells to abundant autoantigen does not only cause deletion but also can result in anergy. Next, we found that mice with a mutation in CNS1 region of Foxp3 that controls pTreg differentiation have a significantly elevated number of anergic CD4^+^ T cells in their peripheral lymphoid organs, supporting the paradigm that anergy precedes CD4^+^Foxp3^−^ T cells differentiation to pTregs, which is further illustrated by a significant overlap between TCR repertoires of Tan and Treg subsets. Finally, we provide evidence that anergy induction helps maintain tolerance to microbiota-derived antigens. We identified specific peptide epitopes derived from the commensal bacteria *Akkermansia muciniphila* (*A. muciniphila*) that induce anergy and support conversion of antigen-specific CD4^+^Foxp3^−^ cells to pTregs. Mechanistically, induction of anergy in CD4^+^Foxp3^−^ cells involved the formation of gap junctions between Tregs and target cells, because the block of intercellular communication between these cells by specifically inhibiting connexin 43 (Cx43) diminished accumulation of anergic CD4^+^Foxp3^−^FR4^+^CD73^+^ cells in the colon. Thus, anergy directly and indirectly, by supporting pTregs formation, contributes to the induction of tolerance to microbial antigens.

## Results

### Recruitment of Tan cells expands the repertoire of pTregs

**Fig. 1:**
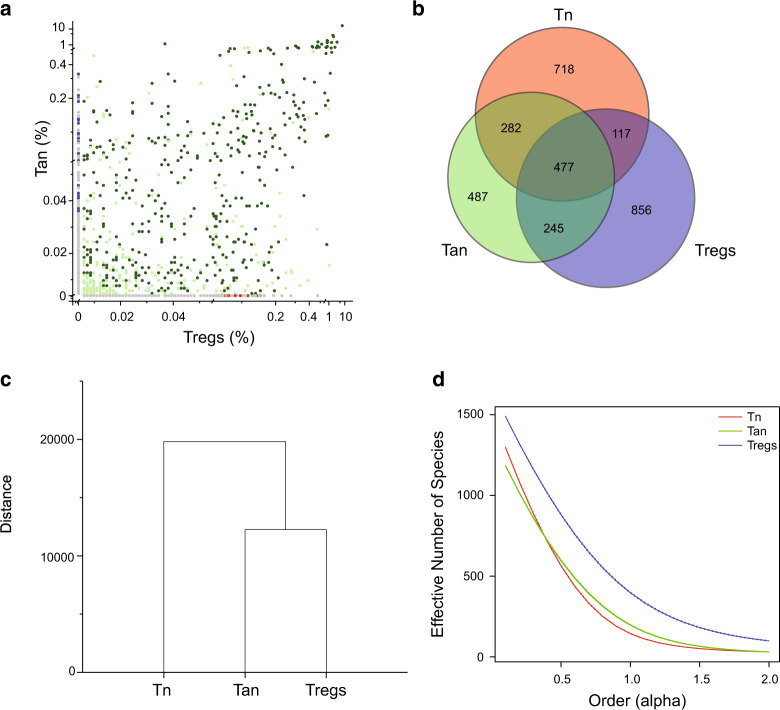
The overlap between the TCRs expressed on Tan, Tn, and Tregs subsets. **a** Frequencies of the TCRs expressed on CD4^+^ Tan and Treg cells from mesenteric lymph nodes of TCR^mini^Foxp3^GFP^ mice and their representation in Tn population from the same organ. Dark green dots mark TCRs shared by all three subpopulations, light green dots depict TCRs shared by Tan and Tregs, and red dots identify TCRs shared by Tregs and Tn subsets. Gray dots on both axes mark TCRs found only on Tregs or Tan cells, whereas blue dots label TCRs shared by Tan and Tn subset. **b** Overlap between all TCRs sequenced from indicated subset of CD4^+^ T cells. Numbers represent quantity of different sequences in indicated populations. **c** Dendrogram showing MII similarity index (overlap) for CDR3α regions of the TCRs expressed by the mLN CD4^+^ Tn, Tan, and Treg cells from TCR^mini^Foxp3^GFP^ mice. **d** Relative diversity index quantified using the effective number of species (ENS) and presented in the form of diversity profiles^43^. Three individual mice were sequenced (23–44 × 10^3^ total sequences per group) and for analysis only sequences present 5× or more were taken under consideration. Results show combined sequences (80–120 × 10^3^ total sequences per group).

We have been investigating the reciprocal relationship between anergic CD4^+^Foxp3^−^FR4^+^CD73^+^ and regulatory CD4^+^Foxp3^+^ T cells, as central subsets regulating peripheral tolerance to tissue and microbiota-derived autoantigens. For this purpose, we used several strains of mice in which T cells that express αβTCRs (TCRs) with restricted diversity (TCR^mini^) naturally differentiate to various functional lineages^11^. In these animals, all TCRs differ only within the sequence encoding CDR3 region of TCRα chain (all TCRα chains incorporate the same Vα2.9 and either Jα2 or Jα26 segments), so high throughput TCRα sequencing can reliably identify the shared or unique clones in different T cell subsets^12^. All T cells in this mouse model use the same TCR Vβ chain (Vβ14Dβ2Jβ2.6) therefore sequencing of the only CDR3 of Vα2 chain can be used to tract different T cell subsets. We found that approximately half (42.6%) of the TCRs abundant on Tregs were also expressed on CD4^+^Foxp3^−^FR4^+^CD73^+^ Tan cells, of which more than 60% were shared by Tn, Tan, and Treg subsets. In contrast, only 7% of TCRs expressed by Tregs were shared exclusively with Tn subset (Fig. 1a, b). Most of these shared TCRs were also expressed on Tn, but only 7% of TCRs expressed by Tregs were shared exclusively with Tn subset (Fig. 1b). These results suggested the scenario in which conversion of most CD4^+^Foxp3^−^ cells to pTregs subset proceeds via an intermediate stage at which these clones express Tan phenotype. Reportedly, expression levels of Nur77^GFP^ reporter by Tregs and by Tan cells appear similar and higher than in Tn cells, suggesting that TCRs expressed by both former subsets have elevated functional affinities for autoantigens^13^. This finding and the relatively high similarity index between Tan and Treg but not Tn and Treg repertoires (Fig. 1c) suggested that most Tregs differentiate from Tan than Tn cells. Further, the conversion of Tan clones could add to the Treg repertoire twice as many new TCRs than reprogramming of Tn clones, as Tregs had the highest diversity of low and high-abundant TCRs from all three CD4^+^ subsets analyzed (Fig. 1d). In summary, these results suggest that most Tn clones undergoing reprogramming to Tregs become anergic before fully converting to Tregs, although direct Tn cells differentiation to pTregs can also occur.

### Foxp3-deficiency limits anergy-induction and enhances CD4^+^Foxp3^−^ T cells resistance to dominant tolerance

**Fig. 2:**
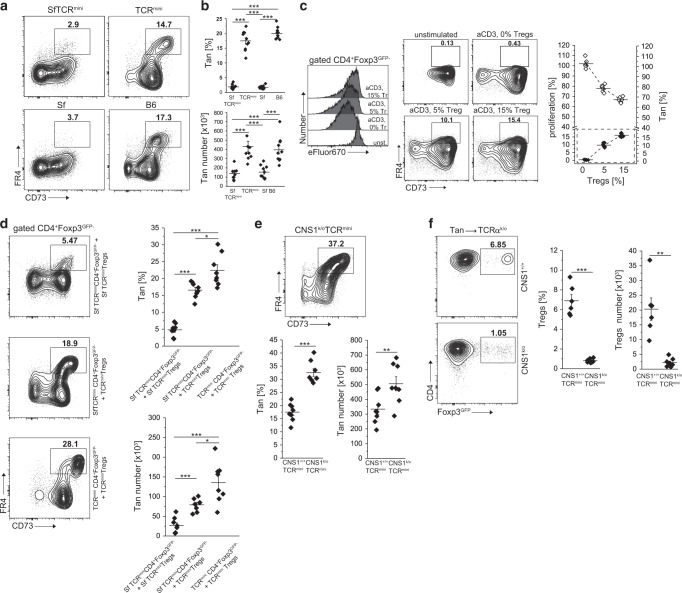
CD4^+^Foxp^3+^ tTregs induce anergy in CD4^+^Foxp^3^^−^ T cells. **a**, **b** Foxp3-deficient scurfy mice have reduced frequency and number of CD4^+^CD44^−^Foxp3^GFP−^FR4^+^CD73^+^ anergic T cells (Tan). **a** Expression of FR4 and CD73 on splenic CD4^+^ cells from healthy C57BL/6 (B6) and scurfy SfC57BL/6 mice that express unmanipulated or restricted (TCR^mini^, SfTCR^mini^) repertoire of αβTCRs. **b** Percentage and a total number of anergic CD4^+^T cells in the spleens from indicated strains (each dot represents an individual animal, *n* = 10 for each type). **c** Ex vivo Tregs induce anergy in naïve CD4^+^ cells. CD4^+^ Tn cells loaded with a proliferation dye eFluor670 were co-cultured with DCs and increasing numbers of Tregs. Typical dilution of eFluor670 on day 4 is shown on a histogram (Tregs abbreviated to “Tr”). Dot plots show the frequencies of FR4^+^CD73^+^ Tan cells within CD4^+^Foxp3^GFP−^ population for each condition. The graph shows the summarized proliferation inhibition and change in Tan frequency (dotted box). One experiment of two is shown. **d** Functional Tregs induce anergy in co-transferred CD4^+^Foxp3^GFP−^ or Sf CD4^+^Foxp3^GFP−^ cells. CD4^+^Foxp3^GFP−^ cells from Foxp3-deficient SfTCR^mini^ or TCR^mini^ mice were adoptively transferred to TCRα^k/o^ mice alone or with Tregs from TCR^mini^ or SfTCR^mini^ mice. Dot plots show proportions of CD4^+^CD44^+^Foxp3^GFP−^FR4^+^CD73^+^ cells in each recipient (each symbol on graphs show frequency or the number of Tan in individual animals (*n* = 8 of each type). Presented FACS data are representative of three experiments. **e** CNS1^k/o^ mice with a defect in pTregs formation have increased numbers of Tan cells. Expression of FR4^+^CD73^+^ on CD4^+^CD44^+^Foxp3^GFP-^ cells in indicated strains (*n* = 8 mice of each type; see also **a** and **b** for comparison). **f** TCRα^k/o^ recipients that received the transfer of Tan from CNS1^+/+^ (*n* = 6) have far more induced pTregs than the same recipients that received Tan cells from CNS1^-/-^ donors (*n* = 8). Dot plots depict typical FR4^+^CD73^+^ Tan frequencies which are summarized on a graph. Statistical significance was calculated with ANOVA with Bonferroni correction (**b**, **d**) or paired Student *t* test (**e**, **f**). *p < 0.05, **p < 0.01, ***p < 0.001.

Conversion of Tan cells to Tregs expands the latter cells’ TCR repertoire, but the role of tTregs and pTregs in Tan recruitment is unknown. Thus, in the following experiments, we studied Tan cells abundance in mice that have intrinsic functional defective all Tregs or only pTregs. The TCR^mini^ mice that lack functional Tregs due to *scurfy* (Sf) mutation in Foxp3 locus (SfTCR^mini^) develop lethal, multiorgan systemic autoimmunity that resembles the disease in original SfC57BL6 mice with *scurfy* mutation^14^. Notably, in contrast to healthy B6 and TCR^mini^ mice, the variant of these strains that harbor Sf mutation in *foxp3* locus had only a few anergic CD4^+^Foxp3^−^CD44^+^FR4^+^CD73^+^ Tan cells in the peripheral lymphoid organs^15^ (Fig. 2a, b). These observations suggested that rapid progression of autoimmunity in mice with Sf mutation may, in part, result from a faulty anergy induction by dysfunctional SfTregs. Ex vivo, the effectiveness of Tregs-induced anergy in CD4^+^Foxp3^−^ T cells increased proportionally to a higher ratio of Tregs to CD4^+^Foxp3^−^ cells (Fig. 2c), suggesting that anergy depends on cell–cell contact between Tregs and target cells^16–18^. Also, in lymphopenic conditions, Tregs-induced anergy in co-transferred CD4^+^Foxp3^−^CD44^−^ (Tn) cells depended on expression of Foxp3 in former subset, because it could not be reproduced by co-transfer of SfTregs, while SfCD4^+^Foxp3^−^ cells resisted stronger induction of anergy than the corresponding CD4^+^Foxp3^−^ T cells (Fig. 2d). Separately, we also found that CNS1^k/o^ mice that lack only pTregs (but have intact tTregs), had increased population of Tan cells accumulating in their peripheral lymphoid organs (Fig. 2e). The last observation suggested that CNS1^k/o^ blocks Tan cells differentiation to pTregs. To directly show that an increase in Tan cells in CNS1^k/o^ mice is caused by these cells impeded conversion to pTregs, we compared differentiation of adoptively transferred Tan cells from CNS1^k/o^ and CNS1^+/+^ mice in lymphopenic B6 TCRα^k/o^ (from now on abbreviated to TCRα^k/o^) host. As shown in Fig. 2f, only Tan cells from the latter but not the former donors’ transitioned to pTregs subset. In summary, these data suggested that Tregs controlled anergy induction in CD4^+^Foxp3^−^ T cells expand the former subset repertoire which helps maintain self-tolerance.

### TCR recognition of the positively selecting self-peptide sustains anergy under lymphopenic conditions

**Fig. 3:**
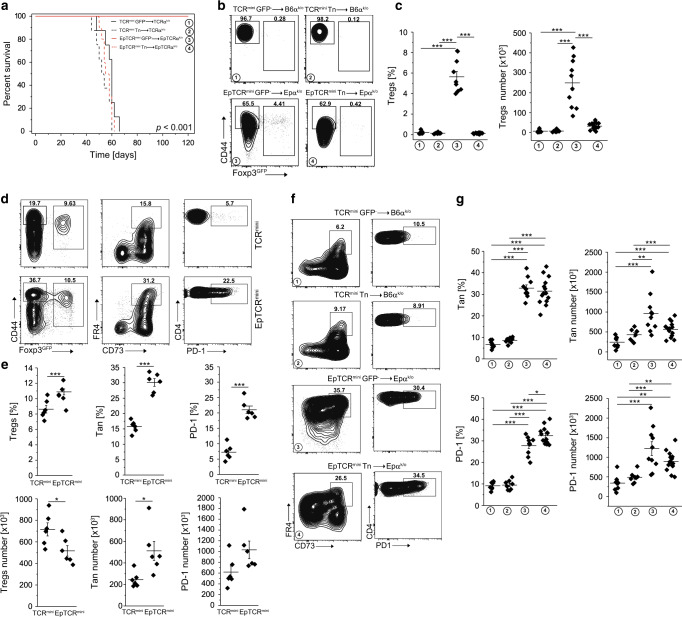
Conversion of Tan cells to pTregs saves lymphopenic recipients from lethal cachexia. **a** Survival of lymphopenic TCRα^k/o^ or EpTCRα^k/o^ mice after transfer of CD4^+^Foxp3^GFP−^ or naïve CD4^+^CD44^−^Foxp3^GFP−^ cells from indicated donors (for CD4^+^CD44^−^Foxp3^GFP−^ and CD4^+^Foxp3^GFP−^ TCR^mini^ donors *n* = 8, for CD4^+^CD44^−^Foxp3^GFP−^ cells from EpTCR^mini^ *n* = 10 and for CD4^+^Foxp3^GFP−^ cells *n* = 14). **b** Expression of Foxp3 and CD44^+^ on transferred cells. **c** Proportions and the total number of Tregs in each recipient. Each symbol represents an individual mouse. **d** Representative analysis of TCR^mini^ and EpTCR^mini^ mice. Frequencies of Tregs (left plots), Tan (middle plots) and PD-1 (right plots) splenic CD4^+^ cells are shown and are summarized in **e** (each symbol shows individual mouse, *n* = 6 of each type). **f** FACS plots show data generated in **a** and **b**. Frequencies of Tan (left) and PD-1 (right) of CD4^+^Foxp3^GFP−^ cells are shown. **g** Summary of data from **f**. Each symbol depicts an individual mouse. Statistical significance was calculated with ANOVA with Bonferroni correction (**c**, **g**) or paired Student *t* test (**e**). *p < 0.05, **p < 0.01, ***p < 0.001.

Although, many Tan cells and Tregs share the same TCRs, the natural peptides that induce anergy and subsequently support the conversion of Tan cells to Tregs are mostly unknown. In particular, the impact of tissue and microbiota-derived peptides remains unclear. Reportedly, the transfer of CD4^+^Foxp3^GFP−^ population that includes Tn and Tan cells to autologous and lymphopenic SPF B6 hosts incited intestinal inflammation and wasting disease (Fig. 3a), whereas the same recipients maintained in germ-free (GF) conditions remained healthy^19^. To further elucidate this phenomenon, we examined an in vivo fate of lymphopenic mice in which class II MHC molecules do not present natural self or microbiota-derived peptides because all A^b^ molecules remain bound with the single, covalently linked (Eα52–68) peptide (EpTCR^mini^ mice)^20^. We found that a transfer of exclusively CD4^+^CD44^−^Foxp3^GFP−^ Tn cells from EpTCR^mini^ mice to lymphopenic EpTCRα^k/o^ recipients resulted in lethal cachexia in <8 weeks (Fig. 3a), demonstrating that a single A^b^Ep complex activated autologous CD4^+^ Tn cells. In contrast, when the EpTCRα^k/o^ recipients received a transfer of the same number of total CD4^+^Foxp3^GFP−^ cells, their health, and survival were unaffected for the duration of the experiment (Fig. 3a). Further analysis showed that upon contact with self A^b^Ep complexes, a fraction of co-transferred Tan converted to Tregs (Fig. 3b, c), and controlled the CD4^+^Foxp3^−^ T cells preventing the onset of wasting disease. Careful examination of TCR^mini^ and EpTCR^mini^ mice revealed that the latter strain had a significantly higher number of Tan and CD4^+^PD-1^+^ cells (Fig. 3d, e). Thus, in lymphopenic hosts recognition of self-peptide(s) by Tan cells promoted tolerance, but the corresponding interactions of Tn cells incited autoimmunity. This last result suggested that transferred CD4^+^Foxp3^−^ cells (Fig. 3a–c) encompassed enough of Tan and CD4^+^PD-1^+^ cells that served as precursors for pTregs formation (Fig. 3f, g). Of note, in the large intestine of TCR^mini^ mice, differentiation of Tan cells to Tregs depended on microbiota-derived antigens, and the number of intestinal Tregs correlated with the number of Tan cells. In contrast, in EpTCR^mini^ mice that express A^b^ unable to present microbiota-derived peptides and GF mice that lack microbiota, the conversion of colonic Tan cells to Tregs was negligible (Supplemenatary Fig. S1A, B). Also, diminished colonic Tregs number in GF and EpTCR^mini^ mice correlated with a smaller Tan cell number (Supplemenatary Fig. S1B), supporting the view that Tan cells and Tregs are reciprocally interdependent. In summary, these results imply that in the lymphopenic recipient’s conversion of Tan cells to Tregs in peripheral lymphoid organs is driven by the body’s self-peptide(s), whereas in the GI tract it is primarily guided by microbiota-derived antigens.

### CD4^+^Foxp3^−^ Tn and Tan differently sense self- and non-self-antigens in lymphopenic conditions

**Fig. 4:**
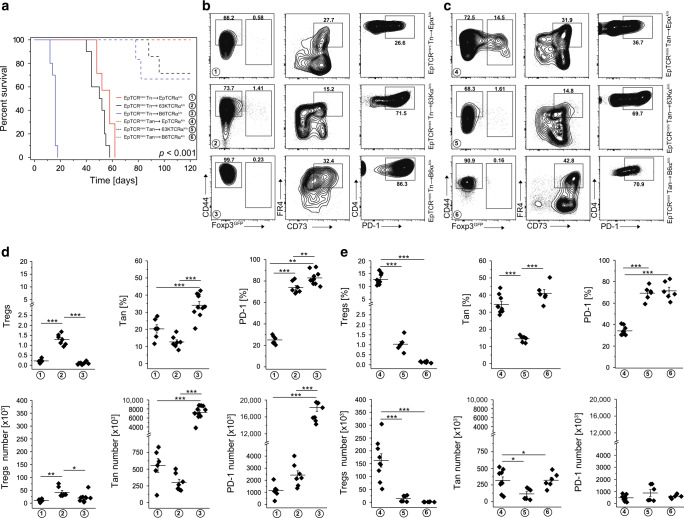
Original selecting peptide suffices to sustains conversion of Tan to pTregs. **a** Survival of indicated host TCR^mini^, EpTCR^mini^ or 63KTCR^mini^ mice receiving adoptive transfer of naïve or anergic CD4^+^ cells from indicated donors (*n* = 6 EpTCR^mini^ Tn->EpTCRα^k/o^, *n* = 7 EpTCR^mini^ Tn->63KTCRα^k/o^, *n* = 10 EpTCR^mini^ Tn->TCRα^k/o^, *n* = 9 EpTCR^mini^ Tan->EpTCRα^k/o^, *n* = 6 EpTCR^mini^ Tan->63KTCRα^k/o^, *n* = 6 EpTCR^mini^ Tan->TCRα^k/o^). Representative FACS analysis of mice from **a**. A typical expression of Foxp3, FR4/CD73, and PD-1 is shown for each host mouse type. **b** represents naïve and **c** Tan cell transfer. Data are summarized in **d** for Tn and **e** for Tan transfers, with each symbol indicating individual animal. Mice expressing symptoms of wasting disease were analyzed when they lost <15% of original starting weight. Statistical significance was calculated by ANOVA with Bonferroni correction. For survival (**a**), the log-rank test was applied. *p < 0.05, **p < 0.01, ***p < 0.001.

In the clinic, local depletion of Tregs followed by a transfer of unfractionated, autologous CD4^+^Foxp3^−^ (containing Tn and Tan) cells have been used to enhance the host’s immune response to infection or tumors^21^. In the following experiments, we examined the fate of transferred CD4^+^Foxp3^−^ cells in lymphopenic recipients that expressed A^b^/self-peptide complexes rarely or frequently recognized by these CD4^+^ cells. For this purpose, we isolated Tn or Tan cells from two different strains of “single A^b^/peptide” mice (expressing exclusively A^b^Ep or A^b^Ep63K complexes) and separately transferred these cells to lymphopenic recipients that expressed A^b^ bound with either many natural self-peptides (TCRα^k/o^), or only single matched or mismatched peptide covalently bound to A^b^ (EpTCRα^k/o^ or Ep63KTCRα^k/o^) (Fig. 4 and Supplemenatary Figs. S2, S3). Most CD4^+^ T cells transferred from donors expressing only A^b^Ep or A^b^Ep63K complexes vigorously responded to natural peptides bound to A^b^ in TCRα^k/o^ recipients, which these cells recognized as non-self-antigens^20^. In contrast, only a few percent of transferred cells recognized A^b^ covalently bound with a mismatched single peptide^20^. Nevertheless, all recipients of Tn cells succumbed to wasting disease within 60 days from the transfer, though host expressing A^b^ bound with wild type self-peptide(s) perished first, followed by recipients expressing A^b^ bound to mismatched or autologous single self-peptide (Fig. 4a and Supplemenatary Fig. S3A). In contrast, all host mice in which A^b^ presented the same single self-peptide as donors survived when injected with Tan cells, but half of the recipients expressing A^b^ bound with different single peptide or many self-peptides perished (Fig. 4a, b and Supplemenatary Figs. S3A, B). Mechanistically, despite that many injected Tn cells upregulated PD-1 inhibitory co-receptor and became anergic, only a few of these cells converted to pTregs (Fig. 4b, d and Supplemenatary Fig. S3B, D), which ultimately led to hosts death. In contrast, ~60% of recipients of Tan cells did not develop cachexia despite their autoantigen(s) differed from donor’s self-peptide (Fig. 4a, dotted blue and black lines and Supplemenatary Fig. S3, dotted red and black lines). The recipients that expressed self-peptides different from donor’s self-peptides and survived had more PD-1^+^ Tan cells (in TCRα^k/o^ recipients) suggesting an enhanced conversion to Tregs (in Ep and Ep63K recipients) (Fig. 4c, e and Supplemenatary Fig. S3C, E). The long-term fate of recipients that received a transfer of CD4^+^ T cells was unchanged regardless if donors expressed A^b^ bound with Ep (Fig. 4) or Ep63K (Supplemenatary Fig. S3) single peptides, suggesting that this outcome was not a phenomenon related to the particular (Ep) peptide. The in vivo data for Ep cells was confirmed in vitro (Supplemenatary Fig. S2). Hence, regardless of the complexity of self-peptides bound to A^b^, in lymphopenic mice donor Tn cells similarly recognize foreign or self-antigens as agonists, whereas to break anergy, Tan cells must encounter the foreign antigen(s).

### Tregs induce anergy to sustain tolerance to microbiota-derived antigens

**Fig. 5:**
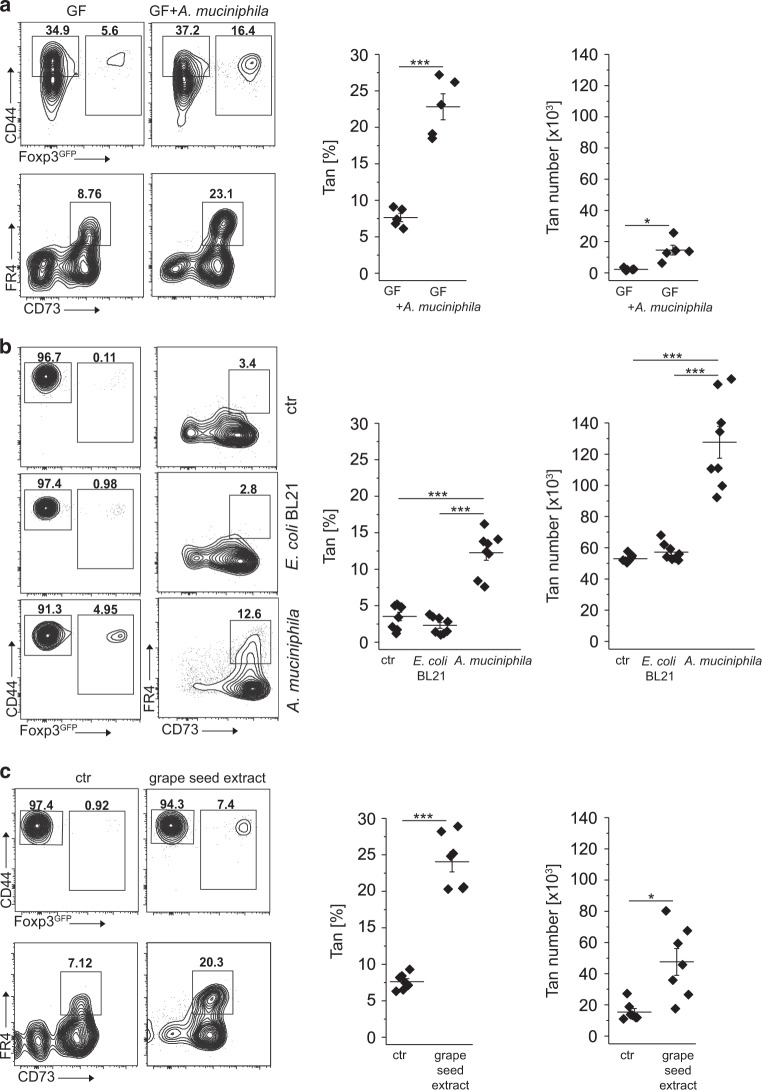
Colonization of GF mice with A. muciniphila supports colonic Tan cells. **a** Colonic Tan cells in 12 weeks old germ-free (GF) mice unmanipulated and mono-colonized with *Akkermansia muciniphila*. Graphs show the frequency and the total number of relevant CD4^+^ cells (each symbol represents an individual mouse, *n* = 5 of each type). **b** Colonization of TCRα^k/o^ with *A. muciniphila* but not with *E. coli* BL21 supports colonic Tan cells and Tregs. Dot plots show representative staining of colonic CD4^+^ cells in TCRα^k/o^ mice first pretreated with antibiotics, then gavaged with PBS (ctr) or indicated bacteria and finally injected with CD4^+^ Tn cells. Expression of CD44, Foxp3, and FR4/CD73 on gated CD4^+^ and CD4^+^Foxp3^GFP−^CD44^+^ cells were examined 8 weeks after adoptive transfer. Symbols on summarizing graphs depict individual animals (*n* = 8 mice in each group). **c** Feeding of TCRα^k/o^ mice with grape seed extract that expands residual *A. muciniphila* increases the number of colonic Tregs and Tan originating from transferred Tn cells. Dot plots show typical Treg and Tan frequencies in PBS-fed control (left) and grape-seed fed mice (right). Graphs show Tan cell frequency and total numbers from individual mice (*n* = 7 mice in each cohort). Plots show representative data of one of three experiments. Statistical significance was calculated by ANOVA with Bonferroni correction. *p < 0.05, **p < 0.01, ***p < 0.001.

Both thymus and peripherally-derived Tregs participate in maintaining tolerance to microbiota-derived antigens, but if anergy in CD4^+^Foxp3^−^ T cells can be induced by specific commensals is currently unknown. We have recently shown that antigens derived from *A. muciniphila* convert CD4^+^Foxp3^−^ T cells to pTregs^22^. Consistently, we also found that colonization of GF mice with *A. muciniphila* besides boosting Tregs number also expanded colonic Tan cells (Fig. 5a and^22^). Likewise, in TCRα^k/o^ mice colonized with *E. coli* BL21DE3 (*E. coli* BL21) or *A. muciniphila* that received Tn cells, only recipients populated with the latter bacteria had many colonic Tan cells (Fig. 5b). Therefore, *A. muciniphila* driven increase in the number of pTregs coincided with the accelerated induction of anergy in CD4^+^Foxp3^−^ cells, which ultimately prevented the onset of wasting disease^22^. Using an alternative protocol, to expand endogenous *A. muciniphila*, we fed TCRα^k/o^ mice with grape seed extract for 3 weeks^22^, then injected these mice with Tn cells and four weeks after transfer analyzed colonic Tan cells in all recipients. As shown in Fig. 5c, feeding with grape-extract but not PBS alone increased the number of colonic Tan cells and Tregs that rescued the host mice from wasting disease^22^. Overall these results suggested that *A. muciniphila* not only supports conversion to pTregs^22^ but also plays a role in the induction of anergy.

### Peptide epitopes from *A. municiphila* induce anergy in colonic CD4^+^Foxp3^−^ T cells

**Fig. 6:**
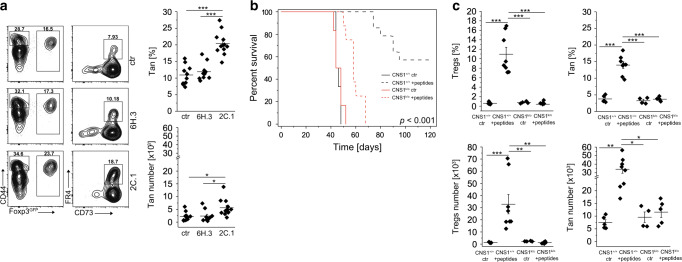
Specific A. muciniphila-derived antigenic peptides induce Tan cells. **a** *A. muciniphila*-derived 2C.1 (*n* = 12 mice) but not control 6H.3 (*n* = 10 mice) peptide induces Tregs and Tan cells. TCR^mini^ mice were gavaged with indicated peptide mixed with cholera toxin or toxin alone (ctr; *n* = 10 mice), boosted i.p. one week later and analyzed on day 14. Plots show representative staining of Tan and Tregs and graphs show data from individual mice. **b** Lymphopenic mice injected with a pool of *A. muciniphila*-derived peptides (2C.1, 2C.9, 6H3, 8H.1) resist wasting disease caused by an adoptive transfer of Tn cells. TCRα^k/o^ mice received pooled peptides with cholera toxin (CT) or CT alone and next day animals were injected with FACS-sorted CD4^+^ Tn cells from CNS1^+/+^ (*n* = 6 ctr and *n* = 14 peptides) or CNS1^k/o^ (*n* = 6 ctr and *n* = 8 peptides). Mice were then gavaged three times with a pool of peptides mixed with CT or CT alone every other day. Then all recipients were i.p. immunized with a pool of peptides adsorbed on an alum every other day for 4 weeks. Ctr-mice received toxin followed by alum only (no peptides). Survival curves for each condition are shown. **c** Summary of Tregs and Tan cells induction for each group of mice. Each symbol in **a** and **c** represents an individual mouse. An experiment was repeated twice. Statistical significance was calculated with ANOVA with Bonferroni correction (**c**) . For survival (**b**), the log-rank test was applied. *p < 0.05, **p < 0.01, ***p < 0.001.

We found that colonization of GF or antibiotic-treated SPF mice with *A. muciniphila* almost doubled the number of colonic Tan cells. This suggests that recognition of bacterial epitopes can induce anergy in specific CD4^+^Foxp3^−^ cells of which a fraction will later differentiate to pTregs (Fig. 5a, b and^23^). To examine if anergy can be induced in Tn cells using a specific *A. muciniphila*-derived peptide, we gavaged TCR^mini^ mice with the 2C.1 or 6H.3 (control) peptides we previously identified to drive colonic Tregs differentiation^23^ mixed with adjuvant (cholera toxin), and after two weeks examined the number of Tan cells in recipients’ colons^22^. As shown in Fig. 6a, treatment with 2C.1 but not 6H.3 peptide or toxin only (ctr) significantly increased the number of these cells in the large intestine, suggesting that the peptide epitope originally described as pTregs-inducer can also facilitate anergy in specific CD4^+^Foxp3^−^ T cells. Alternatively, and not mutually exclusive, Tregs responding to this peptide widen tolerance by inducing anergy in additional Tn clones.

Our previous study identified four *A. muciniphila*-derived peptides (2C.1, 2C.9, 6H.3, and 8H.1) that in mice expressing TCR^mini^ repertoire were recognized by a well-sized pool of CD4^+^ clones^22^. In the following experiments, we asked whether immunization with these epitopes can induce enough of pTregs and Tan cells to suppress an onset of wasting disease in CD4^+^CD45RB^high^ model of colitis. Therefore, we adoptively transferred CD4^+^ Tn cells from either TCR^mini^ mice or TCR^mini^ CNS1^k/o^ donors that lack pTregs, to the lymphopenic TCRα^k/o^ hosts, and then gavaged all recipients with a mix of *A. muciniphila*-derived peptides together with a cholera toxin as an adjuvant. After three rounds of p.o. administration of a peptide pool, we continued with i.p. boosting in which these peptides were adsorbed on alum. As shown in Fig. 6b, most of the recipients of Tn cells from CNS1^+/+^ mice (that also received peptide mix) resisted wasting disease, but the recipients of Tn cells from CNS1^k/o^ donors (receiving or not peptide mix) succumbed to cachexia. We hypothesized that *A. muciniphila* derived epitopes saved hosts from wasting disease by facilitating the conversion of Tn cells directly to pTregs or Tan cells of which some subsequently converted to pTregs. Indeed, host mice that received Tn cells from TCR^mini^CNS1^k/o^ donors lacked pTregs as compared to corresponding mice injected with cells from TCR^mini^ donors also receiving peptide mix, in which proportion and the total number of pTregs significantly increased (Fig. 6c and Supplemenatary Fig. S4B). Importantly, CNS1^+/+^ animals vaccinated with a pool of peptides had the highest numbers of Tan cells and pTregs (Fig. 6c and Supplemenatary Fig. S4B), and the latter cells were readily detected in peripheral blood at week 6 (Supplemenatary Fig. S4A). This therapy also reduced the number of IL-17A and IFN-γ inflammatory T cells and expanded CD4^+^Foxp3^−^IL-10^+^ and CD4^+^Foxp3^+^IL-10^+^ subsets (Supplemenatary Fig. S4C). Overall, these results implied that an anti-inflammatory effect of immunization with a pTregs-inducing, *A. muciniphila*-derived peptides also supports anergy induction.

### Anergy induction ^−^ by CD4^+^Foxp3^+^ Tregs is partially dependent on the expression of connexin 43

**Fig. 7:**
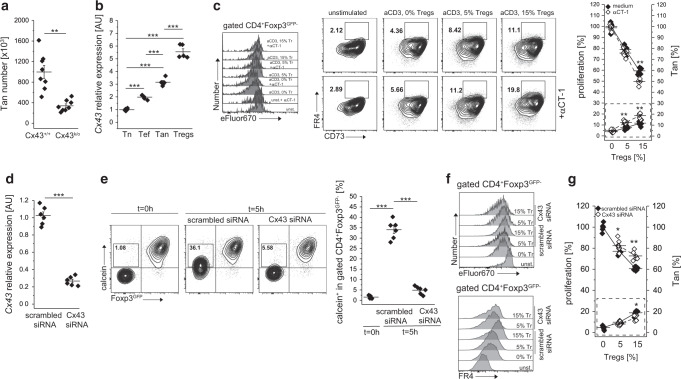
Anergy induction by Tregs involves formation of gap junctions. **a** The expression of connexin 43 (Cx43) impacts the number of anergic CD4^+^ cells in the spleen. Each animal is depicted by an individual symbol (*n* = 8 of each type). **b** Relative levels of Cx43 transcription in naïve (Tn, CD44^−^Foxp3^GFP−^), activated/effector (Te, CD44^+^Foxp3^GFP−^), anergic (Tan, CD44^+^Foxp3^GFP−^FR4^+^CD73^+^), and Treg (Foxp3^GFP+^) CD4^+^ cells examined by real time PCR analysis. Each cell type was analyzed in triplicate. Data from one of two experiments are shown. **c** Ex vivo induction of Cx43 expression by αCT-1 peptide improves Treg-mediated suppression (left *y*-axis) and increases the number of Tan cells (dotted box and right *y*-axis). Representative dot plots and graph of one of two experiments is shown. **d** Relative expression of Cx43 by Tregs transfected with Cx43-targeting or scrambled control siRNA. **e** Implemented Cx43 silencing reduces the transfer of a calcein violet between Tregs and suppressed Tn cells via gap junctions. Box within dot plots show frequencies of Tn cells receiving a calcein. The graph shows data from individual samples. **f** Ex vivo CD4^+^ Tn cells proliferation and FR4 expression upon inhibition by Tregs transfected with Cx43-specific or control siRNA. Tregs abbreviated to “Tr”. The graph on right (**g**) summarizes these data (dotted box shows increase in Tan in response to Tregs). **e**–**g** One experiment of two is shown. Statistical significance was calculated with paired Student *t* test (**a**, **c**, **d**, **g**) or ANOVA with Bonferroni correction (**b**, **e**). *p < 0.05, **p < 0.01, ***p < 0.001.

Although multiple surface receptors and transcription factors have been identified as involved in the induction and maintenance of anergy, the precise mechanism(s) remains unknown. We identified Cx43, a gap junction protein, to be involved in the generation of pTregs and showed that reduced expression of Cx43 on Tregs in NOD mice diminishes these cells suppressor function^24,25^. Reportedly, gap junctions formed by connexin 43 facilitate the transfer of immunosuppressive cAMP that regulates T cell proliferation in response to TCR triggering by impacting their activation^26^. Since Cx43 was attributed to Treg-mediated suppression by us and others^27^, we tested the role of this signaling pathway in anergy induction. We found that mice with a knockout of Cx43 in T cells (Cx43^k/o^) had significantly reduced the total number of splenic Tan cells as compared to Cx43^+/+^ controls (Fig. 7a). Moreover, Tan cells and Tregs had the highest expression of Cx43 (Fig. 7b). Ex vivo treatment of Tregs with αCT-1 peptide, a known Cx43 inducer, and stabilizer, raised expression of Cx43, improved Treg-mediated suppression of CD4^+^Foxp3^−^ and increased the number of Tan cells (Fig. 7c)^25,28^. In contrast, Tregs with siRNA-downregulated Cx43 expression showed an impaired ability to induce anergy ex vivo as compared to their Cx43-sufficient counterparts, indicating that this molecule plays a role in Tregs functions (Fig. 7d–f). Transfection of Cx43-targeting but not scrambled siRNA substantially reduced Cx43 mRNA level (Fig. 7d) and gap junction intercellular communication (Fig. 7e). Importantly, Cx43-siRNA treated Tregs showed a reduced ability to inhibit target cells’ proliferation and to induce anergy (Fig. 6f, g). In sum, these results suggest that Cx43 controlled gap formation influences Tregs induced anergy. Still, the characteristics of CD4^+^ cells susceptible to this mechanism of tolerance remains to be investigated.

## Discussion

In this report, we examined the relationship between Tan and Treg subsets and found that Tan cells constitute a major reservoir of Tregs precursors, which reciprocally by driving Tn conversion to Tregs, support Tan cells sustainability. To document this relationship, we initially examined overlaps between TCR repertoires on Tn, Tan, and Treg subsets. By using the TCR^mini^ mouse model that expresses the semi-diverse repertoire, we performed a TCR sequencing that provided representative data from all studied repertoires. We estimated that approximately half of TCRs expressed by Tregs were shared with the corresponding repertoire of TCRs from Tan cells, suggesting that acquisition of Foxp3 expression and pTreg formation is frequently preceded by anergy induction. These results also implied that two-third of Tan cells recruited to Tregs expressed TCRs already included in Treg the repertoire, whereas the remaining clones expressed new TCRs.

We found that the SfB6 mice had a negligible population of CD4^+^CD44^+^Foxp3^−^FR4^+^CD73^+^ Tan cells in these mice CD4^+^ Tan could not be effectively supported by Foxp3-deficient “wanna-be” SfTCR^mini^Tregs. As Tregs induced anergy involves expression of CTLA-4 and PD-1 inhibitory receptors, SfTregs have likely this function disabled, although Sf mice have more CD4^+^Foxp3^-^CTLA-4^+^PD-1^+^ cells than healthy B6 mice^29^.

In contrast, TCR^mini^CNS1^k/o^ mice with only pTregs deficit, accumulated Tan cells as intermediates stopped on their path to pTregs conversion, suggesting that relatively mild intestinal inflammation observed in these mice may in part depend on the high incidence of Tan cells. Massive accumulation of Tan cells in TCR^mini^CNS1^k/o^ mice showed that tTregs suffice to induce and to sustain Tan subset in vivo. Thus, the functional CNS1 region is non-redundantly required for Tan cells conversion to pTregs, whereas, as reported earlier, the CNS2 region maintains stable Foxp3 expression in pTregs derived from Tan precursors^10^. Since CNS1^k/o^ mice thrive for months before they start manifesting first symptoms of immune responses to microbiota-derived antigens, it is plausible that some of Tan cells support residual tTregs in the induction of tolerance to microbiota-derived antigens partially compensating for pTregs absence.

Reportedly, the average functional affinities of TCRs expressed by Tan cells and Tregs for self MHC/peptide complexes are comparable and higher than corresponding TCRs expressed by Tn cells, based on the expression of Nur77^GFP^ reporter^13^. This observation suggests that strong(er) TCRs signals upon contact with self-peptides support the selection of CD4^+^Foxp3^−^ clones for anergy induction. We found that EpTCR^mini^ mice that express A^b^ bound by the single self-peptide had almost twice as many Tan cells than TCR^mini^ mice. As both these strains are Tregs-sufficient, we speculate that in EpTCR^mini^ high abundance of the same self-peptide across the body enhances anergy induction and sustains Tan long-term persistence. The impact of tissue and microbiota antigens in anergy reversal in lymphopenic mice is also unclear. Co-transfer of Tan and Tn cells from TCR^mini^ mice to autologous and lymphopenic recipients expressing A^b^wt (TCRα^k/o^) resulted in polyclonal activation of all transferred cells, which then caused cachexia. However, when in the lymphopenic host’s microbiota-derived antigens were abolished from A^b^ by tethered single self-peptide, Tan (but not Tn cells) converted to Tregs rescuing most of the recipients. The key factor driving Tan to pTregs conversion was the expression of an identical self-peptide in the donor and host because a substantial number of Tan recipients that expressed non-self-antigens succumbed to wasting disease. Thus, upon lymphopenia response to microbiota antigens reverses anergy, which otherwise is sustained by contact with self-peptides. The study that examined the oral tolerance of OVA-specific T cells transferred into lymphopenic GF mice concluded that commensal enteric bacteria do not enhance the induction of acquired, antigen-specific T cell tolerance^30^. Because the mechanisms of oral tolerance include clonal anergy, deletion of specific T cells, and Tregs formation, the role of microbiota in this model may differ from our experimental settings. Thus, unresponsiveness to natural peripheral autoantigens can be a usual state that precedes T cells deletion or reprogramming to Tregs that prevents disease development^30^. This view also agrees with the reports that antigen doses resulting in tolerance induce both anergy and deletion^31^.

Anergy reversal can also occur in the lymphopenic milieu due to lack of competition with Tregs for relevant self-peptide/MHC II complexes, growth factors (i.e., IL-2 and IL-7), nutrients, and co-stimulatory signals. It commonly occurs in conventional lymphopenic mice, where most Tan downregulate CD73 and FR4 and undergo clonal expansion^32^. In lymphopenic recipients that expressed only A^b^Ep or multiple tissue-derived self-peptides but lacked microbial antigens, transferred Tan cells instead of reverting to effectors, converted to pTregs demonstrating that contact with selecting self-peptide in lymphopenic conditions facilitate such conversion. Strikingly, in A^b^Ep recipients that received a transfer of autologous CD4^+^Foxp3^−^ T cells, their response to abundant self-antigen resulted in anergy without deletion, whereas a similar transfer to hosts expressing A^b^ bound with one or many mismatched self-peptides led to an onset of graft-vs-host disease and minimal induction of Tan cells. Thus, anergy reversal upon T cell lymphopenia is multifactorial and depends on the type of antigen, relative proportions of Tn and Tan in transferred subsets and availability of homeostatic cytokines in lymphopenic hosts. It is uncertain how the anergy depends on intrinsic features of differentiating CD4^+^Foxp3^−^ and if overlapping specificities to common autoantigens or intensity of TCR activation can enhance anergy induction in these cells. Thus, boost of a stable T cell tolerance to self-peptides for more effective treatment of autoimmune diseases while maintaining uncompromised responsiveness to pathogens may require a transfer of autologous, anergic cells naturally converting to pTregs upon contact with self-peptides.

For years, pathogenic bacteria and different viruses have been well known as capable of overstimulating and anergizing a wide range of T or B lymphocytes. For example, Staphylococcus and Streptococcus toxins have been identified as bacterial superantigens inducing anergy, and viral epitopes from mouse mammary tumor viruses, rabies virus (N protein), or human endogenous retrovirus (HERV-K18 env gene)^33^ had a similar tolerizing effect. Similarly, in response to *H. pylori* infection, intestinal Tregs could induce anergy in Tn and activated, pathogen-specific effectors which lead to only mild gastritis and persistent infection^9^. In contrast, the commensal’s epitopes inducing anergy in specific CD4^+^Foxp3^−^ T cells have not been reported, although such antigens have been shown to expand the preexisting Tregs and drive conversion of Tn to pTregs^12,34^. Here we show that Tregs enforced tolerance is supported by microbial epitopes from *A. municiphila* that precondition specific T cells by inducing anergy before some of these cells continue their conversion to pTregs. Whether recognition of these epitopes supports modulations that lead to metabolic reprogramming that stabilize Foxp3 expression and reverse anergy, require further investigation^32^. Yet, blocking of Cx43 reduces the influx of Ca^2+^ and cytokine release by T cells and direct cAMP transfer from Tregs to conventional CD4^+^ T cells via gap junctions (GJ). This contributes to Treg-induced suppression, and connexin proteins as such can directly or indirectly influence the production of cell cycle regulators independently of their channel activities. Our data indicate that Tregs, at least partially, control autoreactive/activated conventional CD4^+^ cell through induction of anergy by using Cx43 which by enhancing anergy and pTregs formation improves the effectiveness of peripheral tolerance. There are two non-mutually exclusive scenarios that can lead to Tan formation. In the first one, activation of naïve T cells by an antigen (self or microbial) upregulates FR4 and CD73 independently of Tregs. In a second scenario preexisting Tregs induce Tan via cell–cell contact, which involves gap junction formation controlled by Cx43. In both cases, Tan cells can subsequently upregulate Foxp3 and convert to pTregs, further accelerating expansion of Tregs number and the repertoire. Thus these two pathways cooperatively ensure the optimal pTregs induction. As Cx43 GJs help sustain clonal expansion of T cells upon effective immune response^35^, optimize T cells activation by enhancing contacts between T and antigen presenting cells^36^, and pleiotropically influence the intestinal inflammation^37^, these published outcomes and results described in this report warrant further investigation of the role of anergy induction to microbiota-derived antigens. Therefore, augmentation of this signaling pathway in Tregs constitutes an attractive tolerogenic immune therapy^38^. The multifaceted aspects of connexin 43-related signaling in cell cycling warrant further investigation^39,40^.

## Materials and methods

Full methods are described in Supplementary materials.

### Mice

The TCR^mini^, A^b^EpTCR^mini^, A^b^63KTCR^mini^ strains, and mice with Foxp3^GFP^ reporter were described^20^. The CNS1^k/o^Foxp3^GFP^TCR^mini^, SfFoxp3^GFP^ (scurfy), and SfFoxp3^GFP^TCR^mini^ were communicated^14,41^. Cx43^k/o^ mice were described^24^. TCRα^k/o^ mice were from Jackson and were bred in our colony. EpTCRα^k/o^ and 63KTCRα^k/o^ mice were described^20^. All experiments were approved by the GSU IACUC Committee.

### Antibodies and reagents

Antibodies and reagents are listed in Table S1.

### Bacteria

*A. muciniphila* was received from Dr. B. Chassaing (INSERM, France) and was grown in an BHI broth in an anaerobic chamber (Coy Lab Products).

### TCR sequencing

TCRVα2 CDR3 regions were amplified as reported^12,14^.

### Real-time PCR

RNA was prepared from FACS-sorted CD4^+^ cells using Qiagen kit and converted to cDNA with SuperScript III kit (Invitrogen). An equal amount of material was run in triplicate using TaqMan (Invitrogen). ΔΔCT method was used for analysis with data normalized to β-actin.

### Tissue preparations, FACS analysis, and cell sorting

Colon samples were prepared and stained as reported^12^. Spleens were disrupted and erythrocytes were lysed with buffered ammonium chloride and washed with HBSS before staining. FACS gating strategy is shown in Supplementary Fig. S5.

### Cell culture

Cells were cultured in CTM media and IL-2 was measured with HT-2 assay^12^. Inhibition assays were done as reported^42^.

### Immunization

Antigenic peptides (ABI Scientific) were mixed with 10 μg cholera toxin (CT; Sigma) at 30 μg/mouse and administered by gavage. Seven days later mice received ip. injection of 15 μg peptide in PBS and were sacrificed on day 14. For vaccination studies, a pool of four *A. municiphila*-derived peptides (25 μg of each/mouse) was mixed with CT and gavaged to mouse. Next day FACS-purified naïve CD4^+^CD44^−^Foxp3^−^ (1 × 10^6^) were intravenously injected into mice. Animals were gavaged every other day for 3 additional times followed by ip. injections three times a week for 4 weeks of peptides adsorbed on alum or alum alone (Alhydrogel; Brenntag) for 5 min before injection.

### Statistical analysis

Data were analyzed with Origin 2017 (OriginLab). Data were presented as the mean values ± SD or survival (Kaplan–Meier) plots. Statistical significance was calculated using ANOVA with Bonferroni correction when more than two groups were analyzed or with paired Student’s *t* test if two groups were analyzed. A log-rank test was applied to compare survival. Differences were considered as significant when *p < 0.05, **p < 0.01, ***p < 0.001.

## Supplementary information

Supplementary material

Supplementary Table 1

## Data Availability

All data needed to evaluate the conclusions in the paper are present in the paper and/or the Supplementary Materials. Additional data related to this paper can be requested from the authors.
