# Supplementary material for: Self and microbiota-derived epitopes induce CD4+ T cell anergy and conversion into CD4+Foxp3+ regulatory cells
Source: Mucosal Immunol. 2020 Nov 2;14(2):443–54. doi: 10.1038/s41385-020-00349-4 (PMC7946630; doi:10.1038/s41385-020-00349-4)
Supplement: Supplementary file 1 — Supplementary material [file 41385_2020_349_MOESM1_ESM.pdf]

Supplementary material

Materials and Methods

Mice

The TCR^{mini}, $A^{b}EpTCR^{mini}$, $A^{b}63KTCR^{mini}$ strains and mice with $Foxp3^{GFP}$ reporter were previously described [20]. The $CNS1^{k/o}Foxp3^{GFP}TCR^{mini}$, $SfFoxp3^{GFP}$ (scurfy) and $SfFoxp3^{GFP}TCR^{mini}$ were also communicated earlier [14,37]. $Cx43^{k/o}$ mice were described [24]. Ultraclean $TCR\alpha^{k/o}$ mice were purchased from Jackson Labs and were bred in our colony. $EpTCR\alpha^{k/o}$ and $63KTCR\alpha^{k/o}$ mice were described earlier [20]. 8-10 weeks old littermate or age-matched mice of both sexes were rederived to ultraclean status at GSU (corresponds to high barrier mice from Jackson laboratories that lack opportunistic pathogens) or as GF strains at UAB. Mice were kept on a sterile chow with a 12-hour light/dark cycle. In some experiments, $TCR\alpha^{k/o}$ mice were pre-treated with antibiotics (ciprofloxacin (0.15 mg/ml), metronidazole (1 mg/ml), streptomycin (2 mg/ml), vancomycin (0.3 mg/ml); all from Sigma) or gavaged with grape seed extract (25 mg/ml; BRI Nutrition) or PBS (22). All presented experiments followed IACUC guidelines and were approved by the GSU IACUC Committee.

Antibodies and reagents

Antibodies and reagents are listed in Table S1.

Bacterial strains

A. muciniphila (ATCC BAA-835) was received from Dr. B. Chassaing (INSERM, France) and was grown in an anaerobe BHI broth (Fisher) in an anaerobic chamber (Coy Lab Products). *A. muciniphila* was grown overnight, washed twice in PBS and 100 μl ($2x10^{8}$ CFU) per mouse was gavaged to 6-8 weeks old mice. *E. coli* BL21 DE3 strain (Invitrogen) was propagated overnight in LB, washed twice in PBS and $2x10^{8}$ CFU was gavaged per a mouse.

TCR sequencing

$TCRV\alpha2$ CDR3 regions were amplified as reported [12,14]. Briefly, FACS-sorted $CD4^{+}$ populations (purity and viability >99%) were used for RNA isolation (RNeasy Mini Kit (Qiagen)). cDNA was made with a primer specific for the TCR Cα region (5′-TCGGCACATTGATTTGGGAGTC-3′) using Superscript III kit (Invitrogen). Illumina sequencing primers were incorporated during 2-step PCR using Accuprime Taq Polymerase (Invitrogen). In the first step, primers specific to the Vα2 (5′-ACACTCTTTCCCTACACGACGCTCTTCCGATCTACAGACTCTCAGCCTGGAGACTCAGCT-3′) and Cα (5′-GTGACTGGAGTTCAGACGTGTGCTCTTCCGATCTTTAACTGGTACACAGCAG-3′) regions of the TCRα chain were used, followed by incorporation of Illumina indexes and adaptors (index 1, i7 adaptors: 5′-CAAGCAGAAGACGGCATACGAGAT*XXXXXXXX*GTGACTGGAGTTCAGACGTGTGCTCTTCCGATC-3′ and index 2, i5 adaptors: 5′-AATGATACGGCGACCACCGAGATCTACAC*XXXXXXXX*ACACTCTTTCCCTACACGACGCTCTTCCGATCT-3′) (italic font marks an Illumina adaptor sequence). Quality of the library was confirmed using Kapa PCR, Qubit and

Fragment Analyzer (High sensitivity NGS fragment analysis) before sequencing with MiSeq Illumina PE250 platform by Georgia Genomics and Bioinformatics Core at The University of Georgia.

Real-time PCR

RNA was prepared from FACS-sorted $CD4^+$ cells using Qiagen kit. DNA-free RNA was converted to cDNA with SuperScript III kit (Invitrogen). An equal amount of material was run in triplicate on Quant Studio 3 Real-Time PCR instrument (Fisher) using TaqMan assays (Invitrogen). A comparative CT ($\Delta\Delta$CT) method was used for analysis with data normalized to β-actin.

Tissue preparations, FACS analysis, and cell sorting

Colon samples were prepared and stained as reported [12]. Spleens were mechanically disrupted and passed through 70 μm filters (Fisher). Erythrocytes were lysed with buffered ammonium chloride and washed with HBSS before staining. FACS gating strategy is shown in Fig. S5. For the detection of intracellular cytokines, cells were stimulated for 4 hours with PMA (50 ng/ml) and ionomycin (500 ng/ml) in the presence of brefeldin A and monensin (each 1:1,000; BioLegend). Surface markers were stained before fixation and permeabilization (Foxp3 kit, eBioscience). T cells *ex vivo* proliferation was assessed by measuring dilution of eFluor670 (eBioscence) dye according to the manufacturer's instruction. Cells were analyzed on day 4 by flow cytometry. FACS analysis was done using CytoFlex (B5-R3-V5) flow cytometer (Beckman) and cells were sorted with SH800 sorter using 100 μm chips with purity >98%, and viability >98% (as assessed by DAPI or trypan blue staining). In some experiments, $CD4^+$ cells were first negatively enriched using MS columns (Miltenyi) to >90% purity before sorting on SH800 instrument. FACS data were analyzed with FlowJo v.10 (FlowJo, LLC).

Cell culture

All cells were cultured in CTM media and IL-2 was measured with HT-2 assay [12]. Inhibition assays were done as reported [38]. Briefly, FACS-purified naïve $CD4^+CD44^-Foxp3^-$ cells were labeled with eFluor670 dye and activated with soluble aCD3 (100 ng/ml) in the presence of GM-CSF-elicited bone marrow-derived dendritic cells (DCs) [22]. The tittered $CD4^+Foxp3^+$ cells were added to selected wells and cells were FACS-analyzed at day 4. In some experiments, αCT-1 peptide (RQPKIWFPNRRKPWKKRPRPDDLEI (American Peptide Company), 30 nM) was added to co-cultured cells. The selected experiments involved the knocking-down *ex vivo* expression of Cx43 in Tregs. For that purpose, the pool of Cx43 or scrambled siRNA (Santa Cruz) was delivered with HiPerfect (Qiagen) to FACS-sorted Tregs which then were incubated overnight with 10 ng/ml of IL-2, washed and co-cultured with tittered number of Tregs in suppression assay. During co-culture Tregs and $CD4^+Foxp3^-$ cells were activated by plate-bound aCD3 (10 μg/ml) and aCD28 (1 μg/ml). Downregulation of Cx43 by siRNA was detected with real-time PCR and by measuring transfer of calcein violet as reported [27]. Briefly, in vitro activated Tregs were loaded with calcein violet (0.5 μM), mixed with activated $CD4^+Foxp3^-$ cells and calcein transfer was assessed 5 hours later by FACS.

Immunization

Antigenic peptides (ABI Scientific, purity >90%) were mixed with 10 μg cholera toxin (CT; Sigma) at 30 μg/mouse and mice were immunized by gavage. Seven days later mice were boosted with ip injection of 15 μg peptide in PBS and sacrificed on day 14. For vaccination studies, a pool of four *A. municiphila*-derived peptides (25 μg of each per a mouse) was mixed with CT and gavages to mice in 100 μl total volume per mouse. One day later FACS-purified naïve $CD4^+CD44^-Foxp3^-$ were intravenously injected into mice. Animals were gavaged every other day for 3 additional times followed by i.p. peptide injections 3 times a week for 4 weeks of peptides adsorbed on alum (Alhydrogel; Brenntag) for 5 min before injection. Control mice received alum only.

Adoptive transfer

FACS-purified naïve $CD4^+CD44^-Foxp3^{GFP-}$cells ($1x10^6$) were adoptively transferred into $TCR\alpha^{k/o}$ mice expressing wild type A^b or A^b covalently linked with Ep or 63K peptides [20]. Animals were monitored for signs of disease (weight loss, diarrhea) and euthanized when met humane endpoint or no later than day 120.

Statistical analysis

Data were analyzed with Origin 2017 (OriginLab). Data were presented as the mean values ± SD or survival (Kaplan-Meier) plots. Statistical significance of differences (p values) was calculated using ANOVA with Bonferroni correction when more than two groups were analyzed or with paired sample Student's *t*-test if two groups were analyzed. A log-rank test was applied to compare survival. Differences were considered as significant when *p<0.05, **p<0.01, ***p<0.001.

For TCR analysis, only CDR3 regions found more than five times in each population per organ and mouse were analyzed. TCRα CDR3 species frequencies were obtained from 2 biological replicates each with 2 technical replicates sequenced both ways. These frequencies were used to determine the number of TCRVα2 CDR3 clonotypes. Statistical significance of the results was performed using Origin Pro (Origin Lab). The analysis was performed using independent samples t-test or a paired sample Student t-test as appropriate. The results were expressed as the mean ± SEM unless stated otherwise. *p≤0.05, **p<0.01, ***p<0.001 as statistically significant. For the comparative analysis of the overlap, a mutual information index (I-index) was used as defined [43]. Diversity was quantified using the effective number of species (ENS) and presented in the form of diversity profiles (see [43] for definitions).

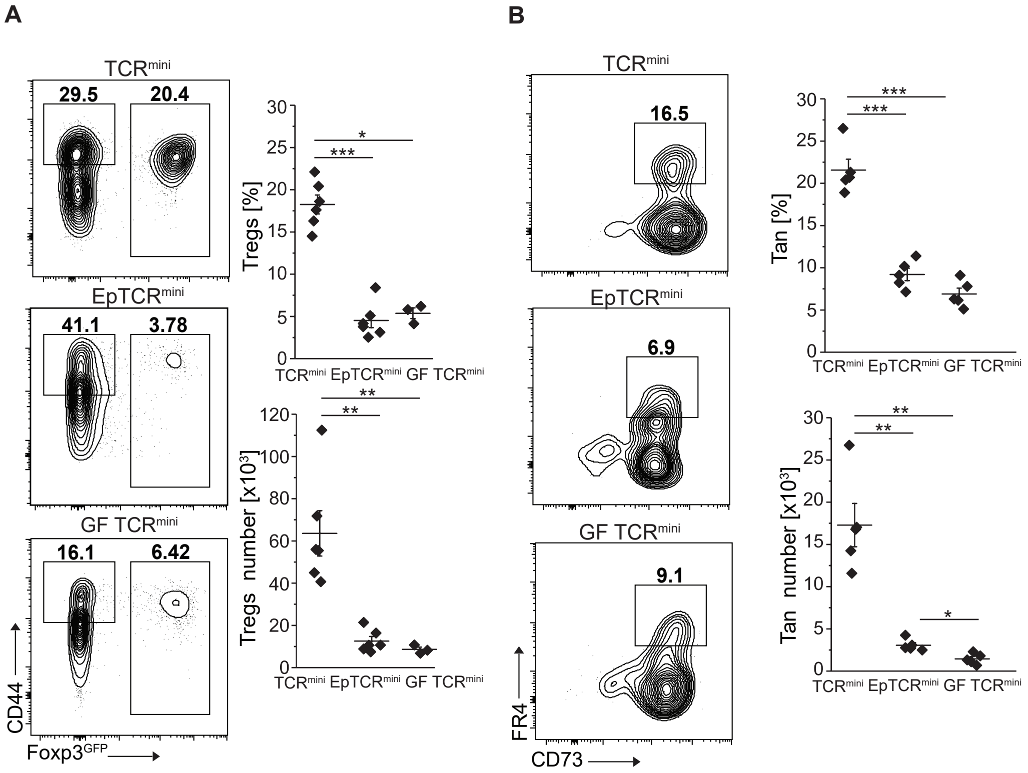

Fig. S1. Colonic Tregs expands upon interactions with commensal microbiota. (A) The inability of presentation of microbial antigens in Ep mice results in a lower number of colonic Tregs. Dot plots depict typical frequencies of colonic Tregs in TCR^{mini} and EpTCR^{mini} mice and percentage and numbers are summarized in a graph (n=6 for TCR^{mini} and EpTCR^{mini} or n=3 for GFTCR^{mini}). (B) Frequencies and numbers of colonic Tan correspond with numbers of Tregs. FR4 and CD73 co-expression are shown in gated CD4$^+$CD44$^+$Foxp3^{GFP-} cells in mice from (B) and summarized on graphs. Typical data of one of two experiments are shown. Data are summarized in graphs. Statistical significance was calculated with ANOVA with Bonferroni correction. *p<0.05, **p<0.01, ***p<0.001.

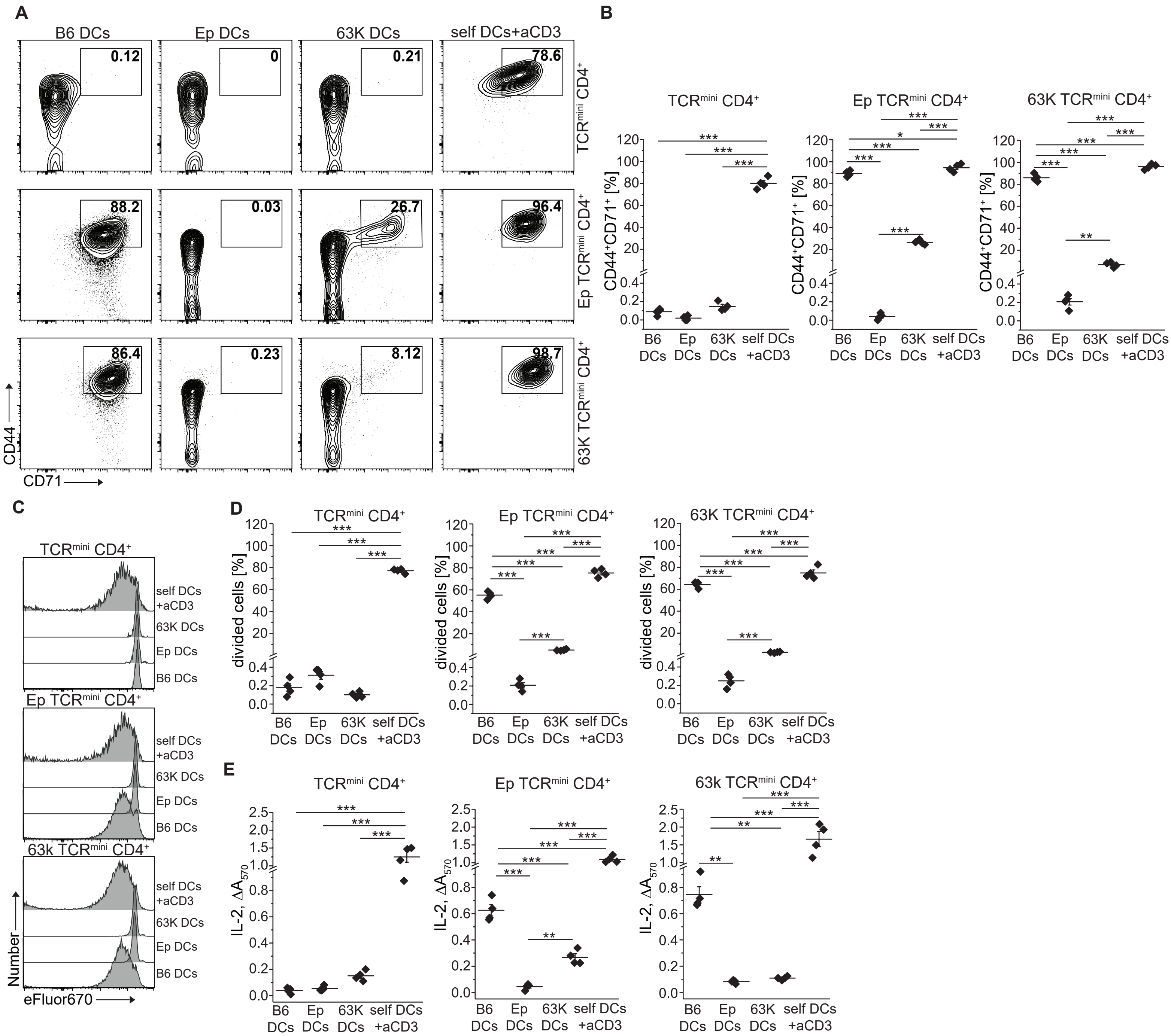

Fig. S2. The magnitude of TCR^{mini}CD4^{+} cells *ex vivo* activation by many wild-type peptides or single peptide bound to A^{b}. (A, B) Expression of activation marker CD44^{+} and proliferation marker CD71^{+} on CD4^{+}Foxp3^{-} cells from TCR^{mini}, EpTCR^{mini} or 63KTCR^{mini} mice co-cultured for 4 days with DCs expressing A^{b}wt, A^{b}Ep or A^{b}Ep63K complexes. Representative staining from one of two experiments is shown, and stimulation with aCD3 MoAb (0.1 μg/ml) was used as a control. (C) The proliferation of activated CD4^{+} T cells (A) measured by eFluor670 dilution; (D) summarizes the data. (E) IL-2 released by activated T cells in indicated conditions measured with HT-2 assay. Each symbol in (B, D, E) represents an individual sample. Statistical significance was calculated with ANOVA with Bonferroni correction. *p<0.05, **p<0.01, ***p<0.001.

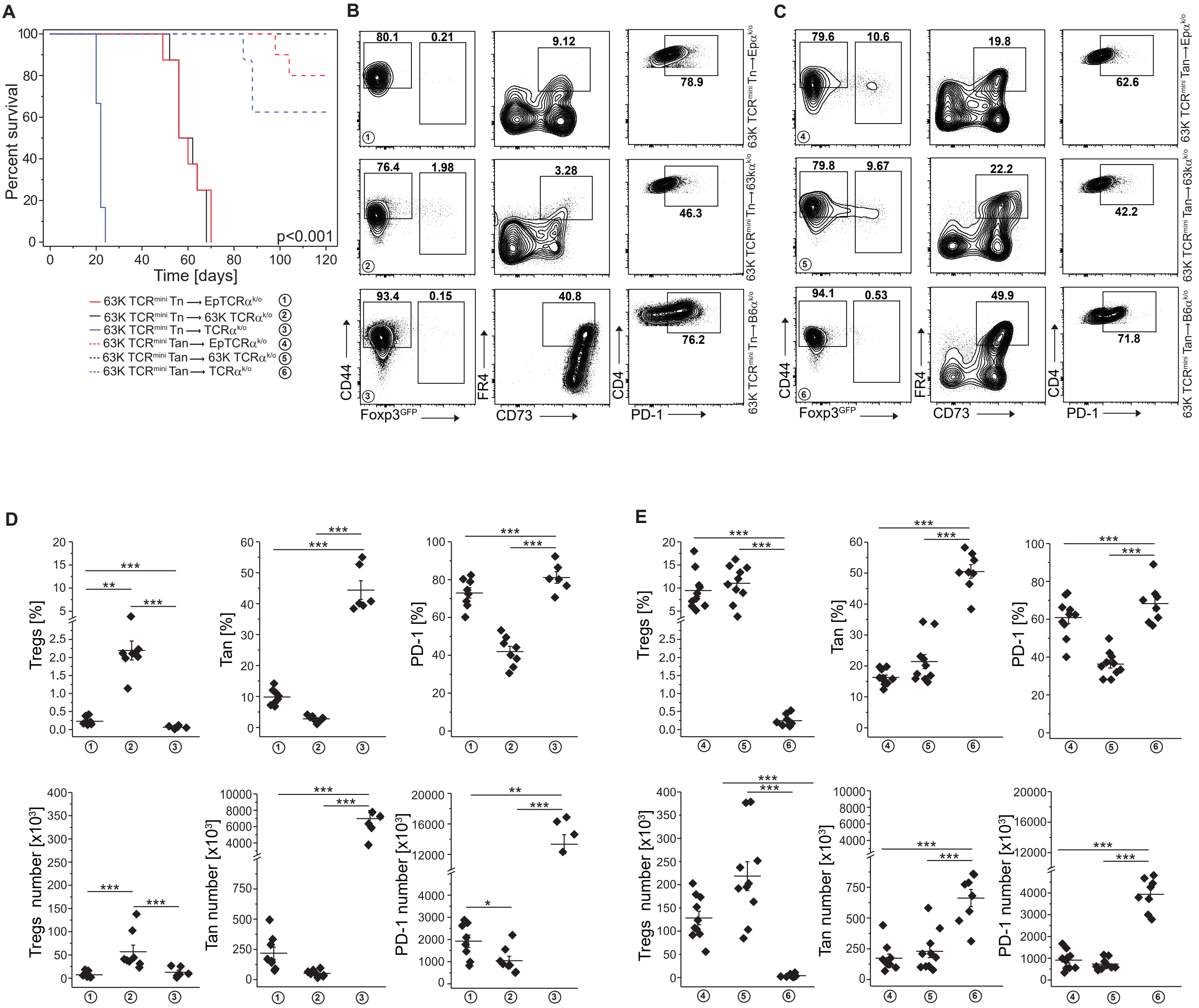

Fig. S3. Anergic CD4^{+} cells frequently convert to pTregs. Presented data corresponds to Fig. 3 but in these experiments transferred CD4^{+} cells were isolated from different donor mice (63K TCR^{mini}). (A) Survival of various lymphopenic TCRα$^{k/o}$ hosts that received transfer of naïve or anergic CD4^{+} cells from indicated donors (n=8 63KTCR^{mini} Tn->EpTCRα$^{k/o}$, n=8 63KTCR^{mini} Tn->63KTCRα$^{k/o}$, n=6 63KTCR^{mini} Tn->TCRα$^{k/o}$, n=10 63K TCR^{mini} Tan->EpTCRα$^{k/o}$, n=10 63K TCR^{mini} Tan->63KTCRα$^{k/o}$, n=8 63K TCR^{mini} Tan->TCRα$^{k/o}$). (B, C) Representative expression of Foxp3, FR4/CD73, and PD-1 by naïve (B) or Tan (C) CD4^{+} cells from experiments shown in panel (A) data from one of three experiments is shown. Data are summarized in (D) for Tn and (E) for Tan transfers, with each symbol indicating individual animal. Statistical significance was calculated by ANOVA with Bonferroni correction. For survival (panel (A)), a log-rank test was applied. *p<0.05, **p<0.01, ***p<0.001

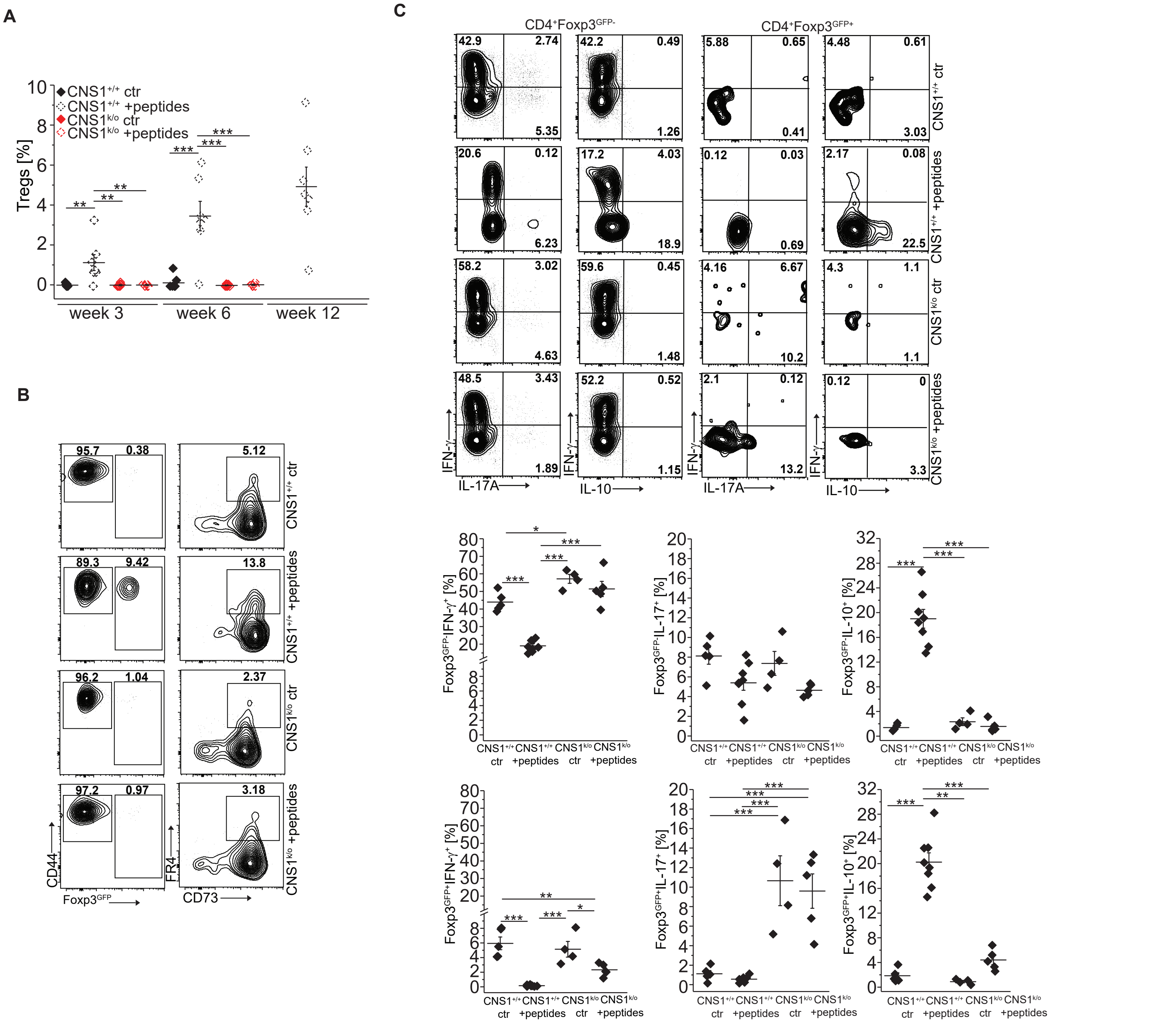

Fig. S4. ***A. muciniphila*-derived mix of antigenic peptides induces pTregs and ameliorate a wasting disease.** (A) Kinetics of pTregs induction in the blood of mice from Fig. 6. Frequencies of colonic CD4$^+$Foxp3^{GFP+} cells are shown for each experimental group. (B) Typical FACS data show expression of Foxp3^{GFP} and FR4/CD73 from representative mice from Fig. 6. (C) Expression of IFN-γ, IL-17 and IL-10 in colonic samples of mice from Fig. 6. Flow cytometry data are shown in gated CD4$^+$Foxp3^{GFP-} and Foxp3^{GFP+} cells. Cytokine data are summarized in graphs on right. Statistical significance was calculated with ANOVA with Bonferroni correction. *$p<0.05$, **$p<0.01$, ***$p<0.001$.

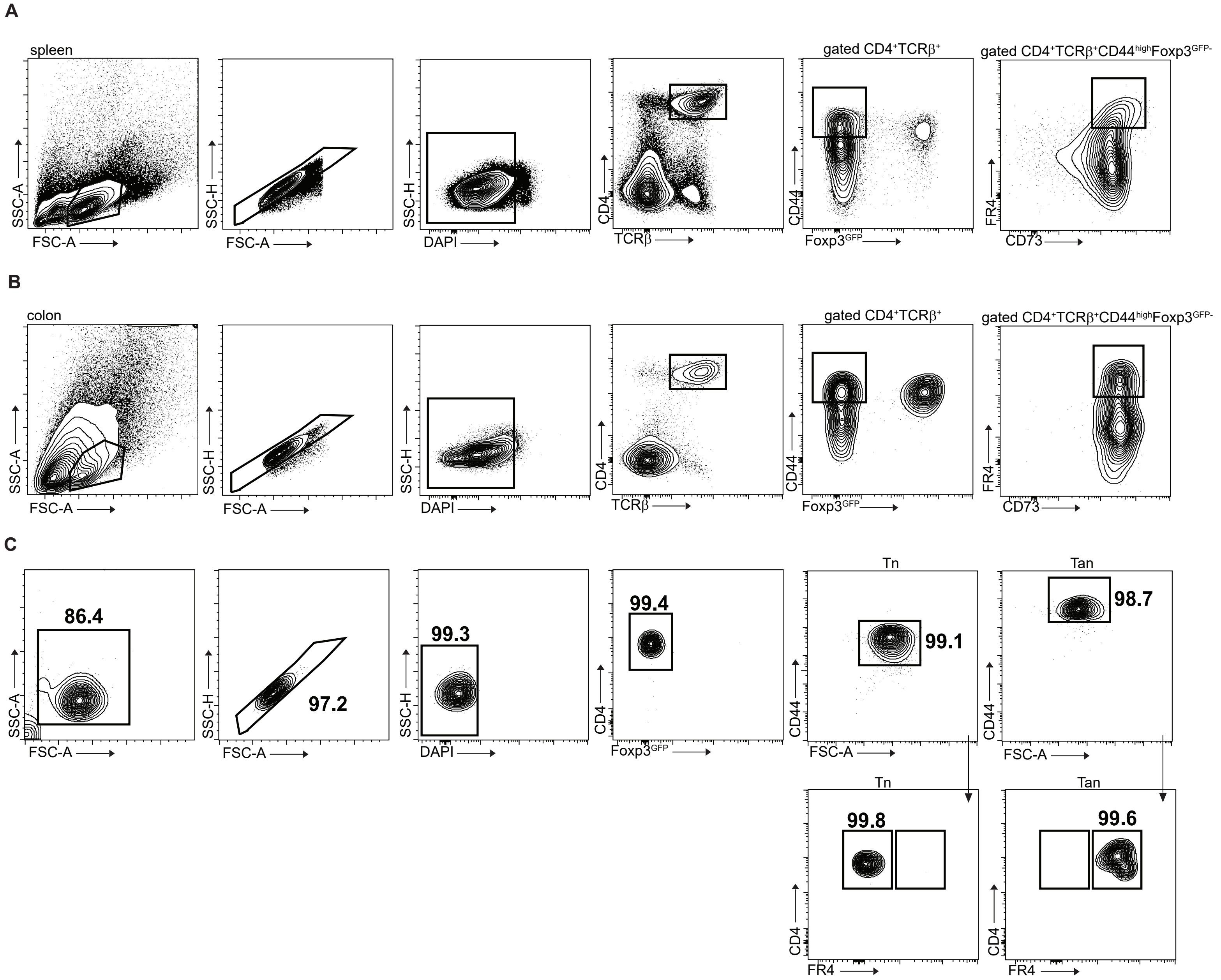

Fig. S5. Gating strategy. (A) Gating for anergic DAPI^{-}CD4^{+}TCRβ^{+}CD44^{+}Foxp3^{GFP-}FR4^{+}CD73^{+} in spleen (A) and colon (B) is shown. (C) Typical FACS-sorting purity of Tn and Tan.
